# Advanced tetra amino (ATA-100) cobalt(II) phthalocyanine-based metallo-covalent organic polymer for sensitively detecting volatile organic compounds

**DOI:** 10.55730/1300-0527.3600

**Published:** 2023-10-11

**Authors:** Günseli GÜNEY, Gülay ALTINDEMİR KAPLAN, Cihat TAŞALTIN, İlke GÜROL

**Affiliations:** 1 TÜBİTAK Marmara Research Center, Materials Technologies, Kocaeli, Turkiye; 2 Department of Chemistry, Faculty of Arts and Science, Yıldız Technical University, İstanbul, Turkiye

**Keywords:** Phthalocyanine (Pc), covalent organic polymer (COP), volatile organic compounds (VOCs), sensor, surface acoustic wave (SAW)

## Abstract

The synthesis and characterization of a novel covalent organic polymer cobalt (II) phthalocyanine (**ATA-100**) including tetra amino group is described for the first time. This covalent organic polymer (COP) is characterized by FTIR, TGA, RAMAN, PXRD, and SEM-EDS. The developed sensor is tested for acetone, ethyl butyrate, *n-*hexane, chloroform, and *n-*butyraldehyde in a range of 80–10,900 ppm. **ATA-100** showed the highest sensitivity for ethyl butyrate. The results have confirmed the possibility of utilizing **ATA-100** COP-based surface acoustic wave (SAW) sensors for a wide variety of applications, including indoor air quality and environmental monitoring of volatile organic compounds (VOCs).

## 1. Introduction

Covalent organic polymers (COPs) typically have high surface areas, allowing for greater interaction with analytes and higher sensitivity in sensing applications. The COPs’ porous structure allows for the selective adsorption of specific molecules, which makes them perfect for use in sensors that need a high level of selectivity [1]. The ability to modify the sensing properties of COPs to target particular analytes is provided by their capability to be synthesized with particular functional groups [2]. These structures are appropriate for severe settings or long-term sensing applications since they are typically stable under a wide range of conditions. Because gas molecule adsorption is reversible, they can be replicated and reused frequently. By synthesizing COPs with various metal ions and organic linkers, a diverse variety of materials with diverse sensing capabilities can be produced [3]. COPs have been demonstrated to have low detection limits for specific gases, making them ideal for use in applications requiring trace gas detection [4]. Devkota et al., for example, discovered methane and carbon dioxide vapors at room temperature in 2018 [5]. Following that, Mirica et al. reported high sensitivity to H_2_S and NO gases in a detection study performed with both Ni-phthalocyanine and Ni-naphthalocyanine in 2018 [6]. The MIL-101(Cr) structure was found to function specifically against pyridine gas in a 2019 study by Haghighi and Zeinali using the QCM sensor, a different type of sensor based on mass [7].

COPs are observed as a promising class of materials for use in sensing applications, such as VOC detection, due to these benefits. The metal used in COPs can have a significant effect on VOC detection. The metal influences the porosity and chemical properties of the coordination polymer, which may affect how well it can adsorb and detect particular VOCs. Which functional group the metal will be interacting with in the analyte gas depends on its coordination number, stability, relationship to other groups that can interact with it, and energy levels. Similarly, the organic linker used in COPs can also play an important role in identifying VOCs. It comes to the fore in determining more than one characteristic of the structure, such as stability, surface area, functionality, and porosity, for a single type of linker or more than one linker coordination polymer used in the formation of the structure. COPs can be tailored to detect a broad range of VOCs with high precision and selectivity by choosing the appropriate metal ion and organic linkers for the target analyte.

Phthalocyanines, a member of the macromolecule class, can be used as organic linkers for COP compounds [6–9]. Nowadays, phthalocyanines are used for VOCs detection in sensor applications [6,10,11]. In addition, COPs are a new concept, and phthalocyanine-based including COPs are limited for the sensor applications [12]. Efficient NO_2_ detection at ppb level of bimetallic phthalocyanine-based COFs (covalent organic frameworks) was demonstrated in 2023 by Xiyu Chen et al. [13]. COPs represent promising platforms for developing new gas sensors based on new functionalization strategies such as compositional and structural control. In this way, high-performance materials for gas detection can be prepared using COPs as templates. With this idea, we have reasoned that the COPs that would arise from this combination could be a material with strong properties for gas sensor applications.

First, starting with 4-nitrophthalonitrile, a cobalt(II) phthalocyanine complex containing the −NO_2_ substitute group was synthesized. After that, the cobalt(II) phthalocyanine compound’s tetra nitro groups at the substitute were converted to the −NH_2_ group in the following step. A 2D COP including cobalt(II) metal was created as a result of the solvothermal reaction between the produced tetra amino substitute group cobalt(II) phthalocyanine compound and 1,3,5-benzenetricarbaldehyde. The sensing properties of **ATA-100** coated on SAW-type sensor bases were investigated under exposure to different concentrations of various VOCs, namely acetone, *n*-hexane, chloroform, *n-*butyraldehyde, ethyl butyrate, and humidity.

## 2. Materials and methods

4-nitrophthalonitrile, cobalt (II) chloride (CoCl_2_), ethylene glycol, nitrogen (N_2_, inert atmosphere), distilled water, diethyl ether, *n*-hexane, toluene, *n-*butyraldehyde, ethyl butyrate, dichloromethane (DCM), methanol (MeOH), disodium sulfide nonahydrate (Na_2_S.9H_2_O), dimethylformamide (DMF), hydrochloric acid (HCl), sodium hydroxide (NaOH), dimethyl sulfoxide (DMSO), benzene-1,3,5-tricarboxaldehyde, tetrahydrofuran (THF), chloroform (CHCl_3_), ethanol (EtOH), and acetone were purchased from Sigma-Aldrich Company Ltd. (Taufkirchen, Germany) and Merck Chemical Industry Co., Ltd. (Germany). Thin-layer chromatography was used to check the purity of the substances at each stage.

The infrared spectra were recorded between 4500 and 600 cm^−1^ using a Perkin Elmer FTIR System Spectrum BX with an attenuated total reflection (ATR) accessory featuring a zinc selenide crystal. Utilizing a LECO TRUSPEC CHN 932 instrument, elemental analysis examinations were performed. On a Bruker Daltonics MicrOTOF, matrix-assisted laser desorption/ionization time-of-flight mass spectrometry (MALDI-TOF MS) measurements were carried out. The MALDI-TOF MS spectra of the compounds were obtained using nitrogen lasers with 50 laser shots, linear mode, and positive ions. The compounds were analyzed in 2,5-dihydroxy benzoic acid (DHB) and dithranol (DIT) MALDI matrices. Raman spectroscopies were conducted via a Renishaw Raman microscope, 2018 model (laser 532 nm (50 mW) and 785 nm (100 mW)). X-ray diffractions (XRD) were performed using Cu Kα radiation at 40 kV and 15 mA and analyzed using the HighScore Plus XRD software. Scanning electron microscopy (SEM) images were obtained using FEI QUANTA 450 model under the following conditions: a 6–10 mm working distance, 0–130 Pa pressure, and voltage of 7–10 kV under low vacuum medium. Thermogravimetric analyses were conducted on a PerkinElmer TGA 8000 at 10 °C min^−1^ in dry air.

In this study, we used the dual-port resonator SAW devices of 433 MHz frequency (SAW Components Dresden GmbH, Germany) mounted on a TO-39 socket. The sensors were placed in temperature-controlled chambers of 4 mL. For the SAW sensor array, the frequencies of the individual sensors were read out sequentially using a multiplexing technique. The seventh uncoated device was used as a reference. The frequency difference between reference and sensor SAWs must be processed instead of the high frequencies of operation.

### 2.1. Preparation of (1)2,(8)9,(15)16,(22)23-tetranitrophthalocyaninato cobalt(II) (1)

A mixture of 4-nitrophthalonitrile (223.3 mg, 1.29 mmol, 4 equiv.) and cobalt(II) chloride (42 mg, 0.32 mmol, 1 equiv.) was refluxed in ethylene glycol (6 mL) for 22 h under inert atmosphere at 150 °C. After cooling to room temperature, the reaction mixture was poured into methanol, and the precipitate was filtered off and washed several times with methanol. It washed with diethylether, water, and *n*-hexane, boiled with toluene in order to separate the dark blue-colored phthalocyanine substance obtained at the end of the reaction from its impurities. The compound was dried in a vacuum at 50 °C.

Yield: 49% (20.7 mg). Mp: >350 ºC. FTIR υ_max_/cm^−1^: 3091, 1613, 1520 (NO_2_ asym.), 1457, 1410, 1330 (NO_2_ sym.), 1252, 1139, 1087, 1044, 945, 905, 846, 819, 759, 728, 676. Anal. Calc. for: (C_32_H_12_CoN_12_O_8_) C 51.15; H 1.61; N 22.37%. Found: C 50.92; H 1.59; N 22.64%. MALDI-TOF MS (m/z) (M_w_: 751.46 g/mol): 756 [M]^+^, 774.23 [M-H_2_O]^+^. RAMAN (20xLP) Raman shift/(cm^−1^): 1608, 1582, 1526, 1413, 1337, 1271, 1231, 1188, 1101, 943, 675, 288.

### 2.2. Preparation of (1)2,(8)9,(15)16,(22)23-tetraaminophthalocyaninato cobalt(II) (2)

Compound **1** (148.80 mg, 0.197 mmol) and Na_2_S.9H_2_O (2.51 g, 10.46 mmol) was refluxed in dimethylformamide (12 mL) for 20 h under inert atmosphere at 80 °C. The reaction mixture was distilled under vacuum at 85 °C until the amount of DMF remained between 2 and 3 mL by distillation method. The substance was precipitated with water, filtered, and washed with water. Firstly, the solid phthalocyanine remaining was washed with 100 mL of 1 M HCl. The green substance turned blue with the acid solvent. Then 1 M NaOH was washed with 100 mL and the base solvent of the substance that was blue was again observed as green [14]. Cobalt phthalocyanine with amino substituents was vacuum dried at 60 °C.

Yield: 72% (107.2 mg). Mp: >250 ºC. FTIR υ_max_/cm^−1^: 3429 (NH), 3333 (NH), 3189, 1606, 1455, 1423, 1345, 1254, 1105, 1034, 992, 875, 820, 750, 616. Anal. Calc. for: (C_32_H_20_CoN_12_) C 60.86; H 3.19; N 26.62%. Found: C 60.62; H 3.20; N 26.64%. MALDI-TOF MS (m/z) (M_w_: 631.53 g/mol): 630,275 [M]^+^. RAMAN (20xLP) Raman shift/(cm^−1^): 1602, 1538, 1455, 1398, 1347, 1253, 1204, 1127, 955, 823, 753, 690, 627, 560, 522, 235.

### 2.3. Preparation of *advanced tetra amino* covalent organic polymer (ATA-100)

Compound **2** (44.5 mg, 0.07 mmol) and benzene-1,3,5-tricarboxaldehyde (22.7 mg, 0.14 mmol) were solvothermal reaction in dimethyl sulfoxide (1.75 mL) and dry ethanol (0.875 mL) for 4 days at 110 °C. During the reaction, 6 M HCl (6 μL) was used as the acid catalyst. Centrifugation was used to separate the solid product of the reaction from the reaction solvent. The substance was washed with dimethyl sulfoxide, chloroform, ethanol, and acetone, respectively, to remove impurities. It was left to dry for a day at room temperature. For activation, it was dried under vacuum at 50 °C overnight.

Yield: 85% (38 mg). Mp: >430 ºC. FTIR (ATR) υ_max_/cm^−1^: 3348, 3216, 3050, 2834, 1693 (O=C-H), 1653 (C=N), 1604, 1514, 1405, 1345, 1314, 1252, 1122, 1083, 950, 934, 885, 826, 771, 751, 678, 615. RAMAN (20xLP) Raman shift/(cm^−1^): 1589, 1541, 1456, 1386, 1326, 1268, 1120, 1093, 991, 827, 751, 687, 240.

### 2.4. Sensor test system

Chemical sensing behavior against polar (acetone, *n-*butyraldehyde, ethyl butyrate, chloroform, and humidity) and nonpolar (*n-*hexane) VOCs was measured for **ATA-100**. Liquid phase VOCs were obtained from Sigma Aldrich Inc., and vapor-phase VOCs were generated from cooled bubblers that were immersed in a temperature-controlled bath that was kept at constant temperature (−12 °C). The concentrations of each gas were calculated based on the Antoine equation [15], varying in the range of 300–11,720 ppm as in our previous paper [10]. Gas mixtures were obtained by an automated mixing system. Saturated VOC vapor was diluted and carried by synthetic air to adjust the gas concentration to the desired amount by using computer-driven mass flow controllers (MKS Instruments Inc., USA) at a constant flow rate of 300 mL/min. Sensors were kept at 25 °C during all measurements. The frequency shift was recorded as a function of time with a sampling rate of 1 s and an accuracy down to 1 Hz. All experiments consisted of repeated exposure to the test VOC (5 min), followed by a flush with pure dry air (5 min) to all molecules, and return to sensor baseline for recovery. Sensors were kept at 25 °C during all measurements. Being one kind of mass-sensitive sensors, the uncoated SAW transducer was used in effort to obtain a frequency reference for sensor response readings [11]. The frequency readings in our experiments were simultaneously obtained by comparing the frequency response of the uncoated SAW transducer with the one coated with the sensing material. Both were exposed to the same analyte flow simultaneously in an array of seven SAW sensors placed in a chamber, one of which was an uncoated reference. The required parameters to understand interaction mechanism between an analyte and the sensor coating the measured compounds of **1**, **2**, and (**ATA-100**) were shown in Table.

### 2.5. Sensor preparation

Compound **1**, compound **2**, and **ATA-100** covalent organic polymer compounds were each prepared separately. One milligram of the compounds was taken and dissolved or dispersed in 0.5 mg of DMSO solvent. This process was carried out for **ATA-100** by applying sonication in an ultrasonic bath for approximately 3 h. The distributed **ATA-100**-DMSO solution was subjected to 1000-fold dilution. The solution was coated on the SAW sensor surface by drop casting under ambient conditions. Compounds **1** and **2** were coated on the sensor surface with a concentration of 2M without dilution. The transducer sensing material area on the surface of the SAW device coated as a drop is approximately 1–2 μL. The resonance frequency shift of SAW sensor devices after coating was measured. Solvent evaporation was performed to achieve a stabilized film surface before finally measuring the coated sensors. Stable evaporation of the DMSO solvent on the sensor surface was expected for one day at room temperature. The coated SAW sensor was then left to dry overnight in a vacuum drying oven at 40 °C.

## 3. Results and discussion

The fundamental design strategy of COPs is focused on offering unique functionalities and active species in their structure. Therefore, the intrinsic performance of the COP can be tuned by selecting suitable organic linkers [16,17]. In other words, the construction of COPs by covalent bonding of the same building blocks in different ways can create an effective sensing material specific to the desired sensor application area. **ATA-100** was synthesized by the condensation reaction of Co(II) tetra amino phthalocyanine (compound **2**) and benzene-1,3,5-tricarboxaldehyde organic linker under solvothermal conditions as shown in Scheme. The synthesized **1**, **2**, and **ATA-100** compounds underwent a variety of characterization techniques.

**Figure 1:**
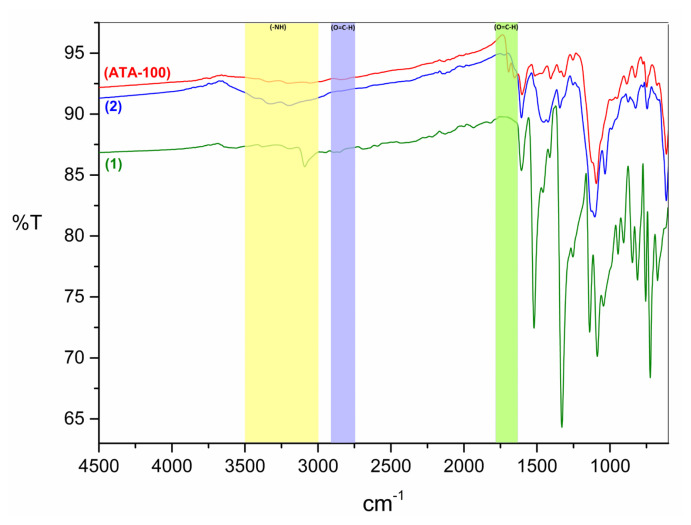
FTIR spectrum of compounds **1**, **2**, and **ATA-100**.

All three substances have spectra in the FTIR analysis, as shown in Figure 1, and the major differences are highlighted in color. The most important parameter for compound **1** FTIR analysis was a nitrile peak in 4-nitrophthalonitrile material around 2240 cm^−1^. The asymmetric stretching peak of the substituted group, −NO_2_, is at 1520 cm^−1^, the symmetric stretching peak was observed at 1330 cm^−1^, and the stretching vibration peak of the link between C-N was at 846 cm^−1^ (Figure S1). Compound **2** with the −NH_2_ substitution group was generated by reducing the −NO_2_ group in the structure of compound **1** with the reduction agent Na_2_S. The phthalocyanine ring’s distinctive peaks were intact, with only minor alterations noted. The −NO_2_ group peaks at 1520 cm^−1^ and 1330 cm^−1^ in the compound **1** disappeared. The stretching peaks of the primary amine (NH_2_) group seen at 3429 cm^−1^ and 3333 cm^−1^ are the peaks, indicating that the reduction occurred. Besides, in plane bending vibration of −NH_2_ group and out plane deformation vibrations of its were observed at 1606 cm^−1^ and 992 cm^−1^, respectively (Figure S2). As the last, for **ATA-100**, synthesized by compound **2** and benzene-1,3,5-tricarboxaldehyde via Schiff-base reaction, the peaks at 3348 cm^−1^ attributed to the N-H stretching in amino group, and the peaks at 1653 cm^−1^, 1514 cm^−1^, and 1450 cm^−1^ are attributed to the C=C and C=N stretching vibration of phthalocyanine, respectively. In addition, the peaks at 1083 cm^−1^ and 751 cm^−1^ are attributed to the vibration and characteristic band of phthalocyanine macrocycles (Figure S3) [18]. The second important parameter for the elucidation of the structure is the absence of strong carbonyl moieties of the aldehydes (O=C-H) peaks at 2871 cm^−1^, 2852 cm^−1^, and 1692 cm^−1^ belonging to the benzene-1,3,5-carboxaldehyde group. However, since two aldehyde groups are needed for the repetition of the polymer and will cause steric hindrance of third aldehyde groups, one aldehyde group in the structure remained as a functional group in the coordination polymer without reacting. For this reason, the peaks observed at 2834 cm^−1^ and 1693 cm^−1^ in **ATA-100** spectrum are attributed to the carbonyl (O=C-H) group in the aldehyde structure (Figure S4). There is a serious decrease in the intensity of these peaks compared to the observed peak of benzene-1,3,5-carboxaldehyde. Looking at the other peaks in **ATA-100** structure, these peaks constitute the phthalocyanine structure’s distinctive peaks.

**Figure 2:**
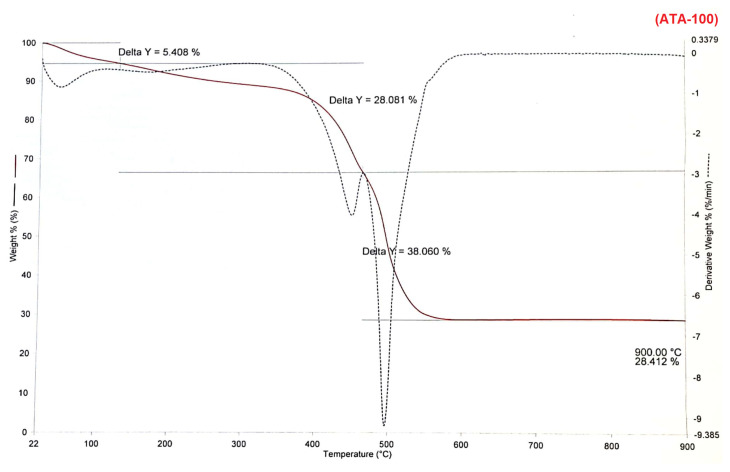
TGA spectrum of **ATA-100**.

Thermogravimetric analysis (TGA) is also performed in air atmosphere to examine the content of compounds **1** and **2** and the stability of **ATA-100**. TGA performed in air was examined for each synthesized compound. Compounds **1** and **2** were found to be stable up to a temperature of about 250–350 °C, with any minor weight losses due to humidity in the structure (Figures S5 and S6). Analysis of **ATA-100** reveals its excellent thermal stability up to 450–500 °C with only slight weight losses (5%); this loss can be attributed to the removal of entrapped solvents (Figure 2). It demonstrates that after decomposition, the sample weight stayed at around 28.41% and that the residue was cobalt tetroxide (Co_3_O_4_). The leftover Co_3_O_4_ allows for the production of 71.59% by weight of COP that is present in **ATA-100**. Because of its thermal stability, **ATA-100** is an excellent foundation for subsequent study in application areas.

**Figure 3:**
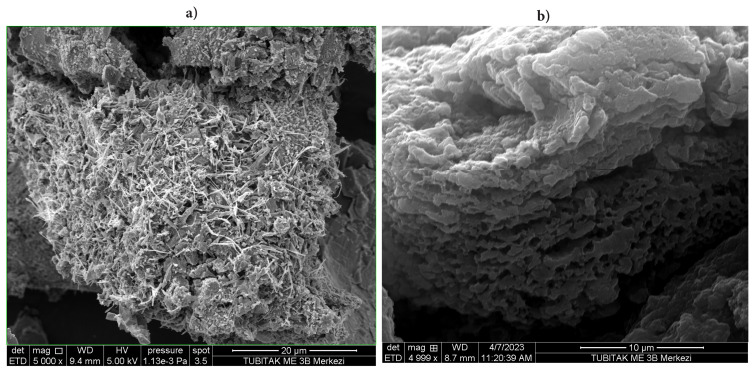
SEM images of **(a)** compound **2** and **(b) ATA-100**.

SEM measurements provided more information regarding the morphology of the produced samples. Compound **2** had an ordered arrangement and a needle morphology. The morphology of **ATA-100** structure is seen as stacked at 10 μm. It can be interpreted from this image that it is 2D material. It is thought to have a microporous structure, and because of its small pore structure, phthalocyanine unit aggregation may be effectively prevented, and the microporous structure can be used effectively in sensor applications. SEM-EDS analysis was performed by gold plating (Figure 3).

Elemental mapping by energy dispersive spectroscopy (EDS) exhibits the elements C, N, O and Co (Figure S7), which is consistent with the composition of **ATA-100**.

**Figure 4:**
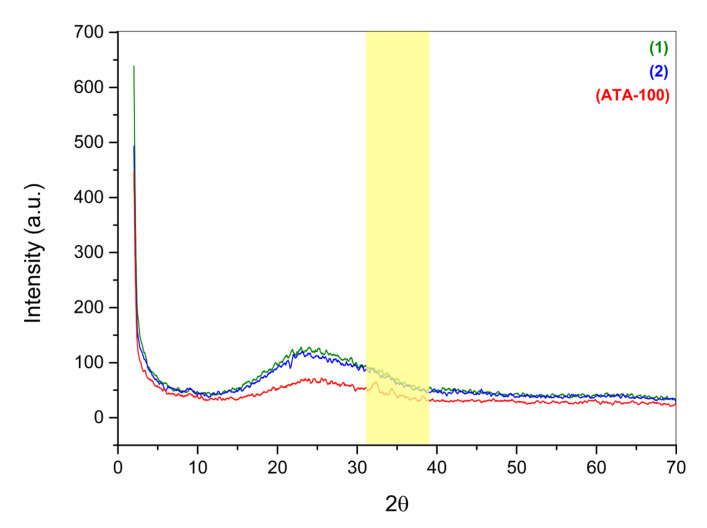
PXRD spectrum of compounds **1**, **2**, and **ATA-100**.

Compounds **1**, **2**, and **ATA-100** powder X-ray diffractometry (PXRD) analyses were also performed and compared to similar research in the literature (Figure 4) [19]. The experimental PXRD 2ϴ = 14.14, 19.46, 24.64, 32.32, 34.38, 38.28 degree peaks were observed to match the computationally determined diffraction peaks in the literature [19]. It was determined that the 2ϴ = 9.02, 21.34, 22.04, 23.06 values found in the PXRD analysis for compound **2** are different from the results of the PXRD analysis performed for **ATA-100** (Figures S8 and S9). The broad peak indicates the presence of the samples amorphous fraction. Due to the fact that **ATA-100** COP is based on phthalocyanine, amorphous structure was seen in it, and sharp peaks were determined to be distinctive among wide peaks (Figure S10). The peaks between 2ϴ = 30–40 observed in the XRD spectrum of ATA-100 belong to the cobalt metal, and as the COP material moves away from the amorphous structure according to the phthalocyanine structure, the peaks in this range can be observed. The determined peaks were found to be compatible when compared with the computationally determined PXRD peaks [19,20].

**Figure 5:**
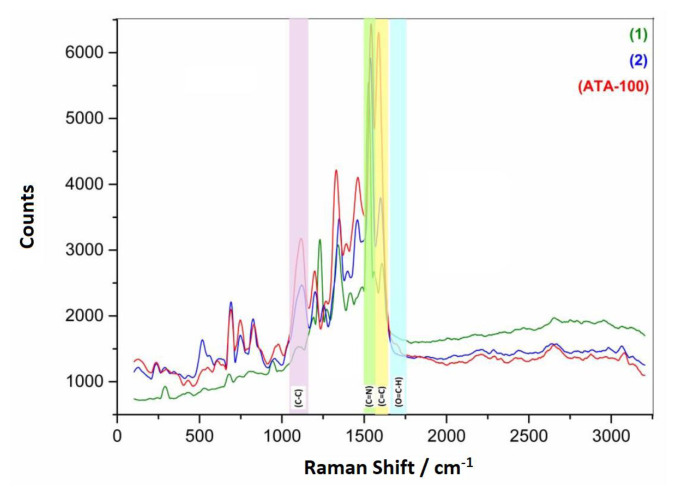
Raman spectrum of compounds **1**, **2**, and **ATA-100**.

The Raman bands assigned to compound **1** (Figure S11), compound **2** (Figure S12), and **ATA-100** exhibit comparable characteristic peaks with (Figure 5) showing the spectra from powder. When examine the Raman spectrum of **ATA-100**; Aromatic C-C stretching peak of benzene group at 1589 cm^−1^, pyrrole stretching, isoindole ring stretching and displacement of the C–N–C bridge bond related to central cobalt metal ion of phthalocyanine molecule are observed at 1541 cm^−1^, 1456 cm^−1^, 1386 cm^−1^, 1326 cm^−1^, and 1120 cm^−1^. In addition, C-H bending at 1093 cm^−1^, the peak of the C-C bond of benzene breathing is 991 cm^−1^, the peak of the vibration of the C-N-C bonds at 827 cm^−1^ is seen [21,22]. The vibration of the bonds being with metal group of nitrogen atom in the phthalocyanine structure (N-Co-N and N-Co) is seen at 240 cm^−1^.

Compound **2** and **ATA-100** are investigated comparatively, it is observed that the intensity of the peaks at 1127 cm^−1^ and 1347 cm^−1^ of the C-C group of the benzene ring in compound **2** increased in **ATA-100**. At the same time, a severe increase is observed in the peak belonging to the aromatic C-C group at 1589 cm^−1^. These conditions can be attributed to the increase in the rate of conjugation and benzene ring presence in the structure, together with the aldehyde group binding to the structure and forming a reproducible COP (Figure S13). The new C=N bond generated as a result of the Schiff base reaction is attributed to be responsible for the considerable rise in the strength of the C=N peak at 1541 cm^−1^ in **ATA-100** [23]. As a result of the reaction, an aldehyde functional group in the structure of benzene-1,3,5-tricarboxaldehyde remained as a carbonyl group rather than forming an imine bond. The newly generated peak at 1695 cm^−1^ in **ATA-100** is attributable to the peak of this status indicator.

### Sensor response

The performance of the sensing layer is directly impacted by the structural and chemical characteristics of the sensing layer, which are widely recognized to have a significant effect on the sensor qualities. Moreover, molecules’ spatial positions are significantly influenced by the structure of compounds. Sensitivity values must be meticulously examined in order to correctly estimate the interaction mechanism. The thickness of the film and the material’s conductivity can affect the response of the SAW sensors [10]. As a result, sensitivity values are contrasted to define selectivity and the quantity of response to the analyte in order to accurately assess the sensing capacities of the molecules.

**Figure 6:**
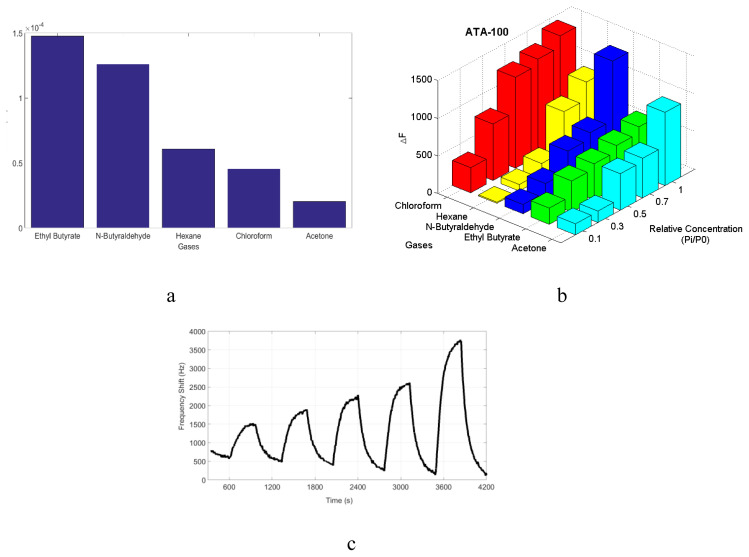
(**a**) Sensor sensitivity values for **ATA-100**, (**b**) sensor responses for **ATA-100** are shown for different VOCs (acetone, ethyl butyrate, *n-*butyraldehyde, *n-*hexane and chloroform), (**c**) sensor response versus time for ethyl butyrate.

In this study, we observed that **ATA-100** showed the highest sensitivity value towards ethyl butyrate and *n*-butyraldehyde among all analyte materials tested of **ATA-100** (Figure 6). The reason of this is that the dielectric constant values seen in Tablo affect the detection mechanism of **ATA-100**. On the other hand, molecular weight is another expected effect on sensor response. Considering that the SAW-type transducer is mass-sensitive, it becomes clear that we must also consider molecular weight to explain the interaction. The sensing phenomenon is based on the assumption that the sensor surface is one of the small sensing areas and that a molecule is detected in each area. Therefore, it can be concluded that the highest sensitivity should be for ethyl butyrate and *n-*butyraldehyde.

In addition to all this on the intermolecular interactions, cavities called intermolecular gaps into which gas molecules can settle may form, and gaseous analytes can adsorb into these intermolecular gaps, i.e. space between each other of **ATA-100** molecules and the molecular structure of **ATA-100**. For a better comparison, the responses of different vapor pressures’ relative concentrations pi/p0i are used, where pi is the actual analyte concentration and p0i is the saturation vapor pressure at room temperature (Figure 6a).

From the perspective of intermolecular interactions, adsorption of chloroform is considered to occur as a result of interactions between the partial positive charge on the H atoms of the chloroform molecule and the polar oxygen (C=O) group that is present on the benzene-1,3,5-tricarboxaldehyde in **ATA-100** COP. The same situation can be considered for *n-*butyraldehyde. The hydrogen atom of *n-*butyraldehyde is partial positive due to C=O atoms present in the molecule (Figure 6b).

The sensor responses are linearly increasing with concentration. A linear fit to the response data (frequency shifts during analyte exposure from baseline) versus concentration was made, and the slope is the sensitivity (Hz/ppm) (Figure 6b).

Compound **1** and compound **2** are measured for acetone, ethyl butyrate, *n-*hexane, chloroform, and *n-*butyraldehyde. However, **ATA-100** has the best sensor response according to compounds (Figure 6c).

**Figure 7:**
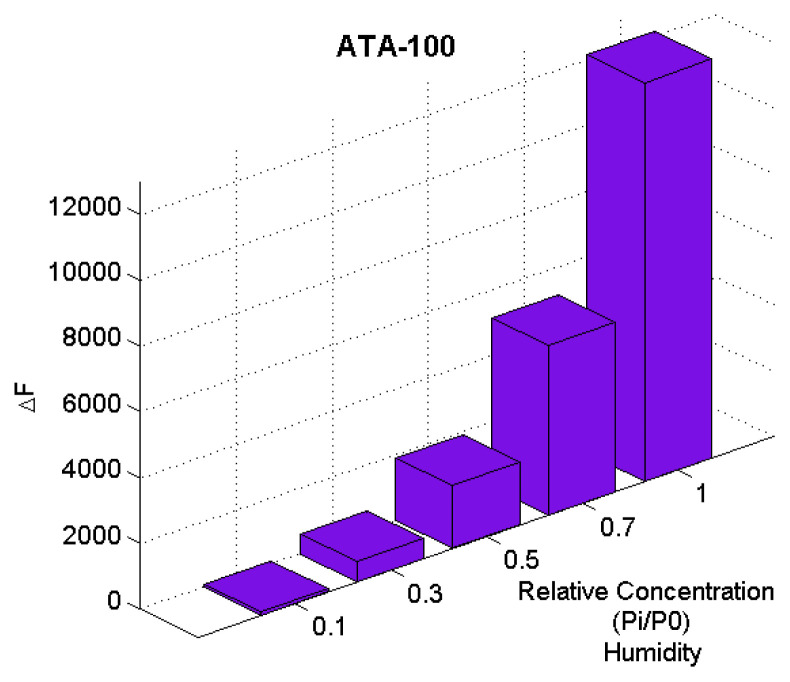
Sensor responses for **ATA-100** are shown for humidity at different concentrations.

**ATA-100** molecule, which has the strongest sensitivity value to the *n-*butyraldehyde analyte, is observed to exhibit high sensitivity to humidity (Figure 7).

## 4. Conclusion

Covalent organic polymers (COPs) have been derivatized with various organic linkers and gained new properties. In this article, the macrocyclic compound phthalocyanine is used as an organic building block for COP, which is one of the most important material classes in recent years. Syntheses of compounds **1** and **2** with phthalocyanine structure and **ATA-100** as COP were performed and characterized by using FTIR, TGA, Raman, PXRD, and SEM-EDS. Compound **2** and benzene-1,3,5-tricarboxaldehyde were used to obtain **ATA-100**. It was studied by comparing it with five different VOCs at different concentrations to investigate their detection properties. The detection properties of compounds and **ATA-100** coated on SAW-type sensor bases were investigated by exposure to different VOC concentrations such as acetone, ethyl butyrate, *n-*hexane, chloroform, and *n-*butyraldehyde. Gas detection measurements were carried out at room temperature in a dry-air ambient atmosphere. However, **ATA-100** has the best sensor response, according to compounds **1** and **2**. **ATA-100** showed the highest sensitivity for ethyl butyrate in a range of 80–500 ppm, which could result from dielectric constant values between the analyte and sensing molecule. The relative humidity detecting capability of the **ATA-100** was tested at relative concentrations between 0.1 and 1. In future work, this sensor will also be investigated using Raman analysis to better understand the detection mechanisms and chemical sensor properties versus the detection of VOCs.

## Supplementary Information

Figure S1 FTIR spectrum of compound **1**.

Figure S2 FTIR spectrum of compound **2**.

Figure S3 FTIR spectrum of **ATA-100**.

Figure S4 FTIR spectrum of benzene-1,3,5-tricarboxaldehyde.

Figure S5 TGA spectrum of compound **1**.

Figure S6 TGA spectrum of compound **2**.

Figure S7 EDS spectrum of **ATA-100**.

Figure S8 PXRD spectrum of compound **1**.

Figure S9 PXRD spectrum of compound **2**.

Figure S10 PXRD spectrum of **ATA-100**.

Figure S11 Raman spectrum of compound **1**.

Figure S12 Raman spectrum of compound **2**.

Figure S13 Raman spectrum of **ATA-100**.

## Figures and Tables

**Scheme:**
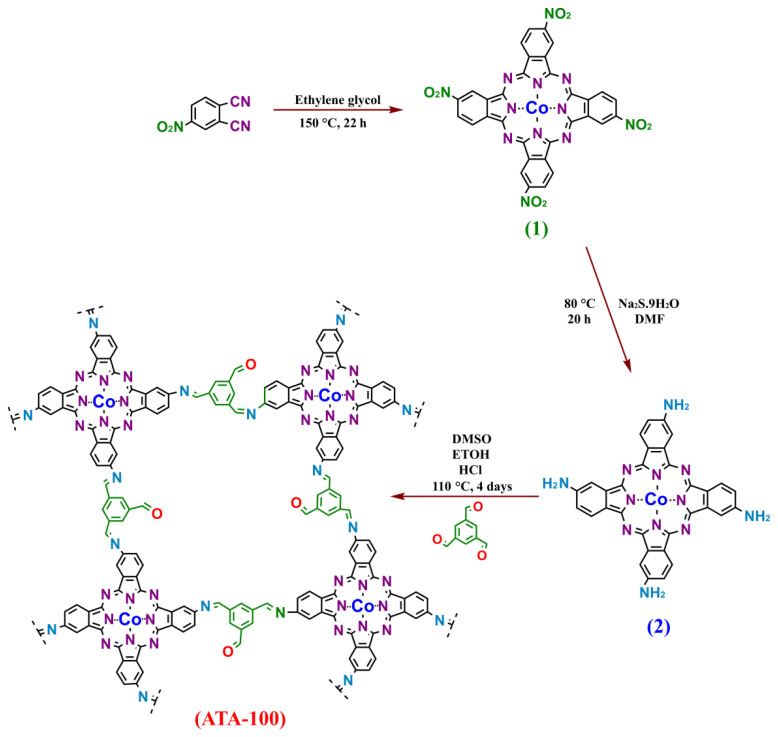
The schematic illustration of the synthesis process of **ATA-100**.

**Table:** Properties of analytes: dielectric constant, dipole moment, the tested concentration range and molecular weight.

Analytes	Molecular weight (g/mol)	Dielectric constant (ɛ)	Dipole moment (μ)	Concentration (ppm)	
				Min.	Max.
**Hexane**	86.18	1.89	0.08	580	5.800
**Chloroform**	119.3	4.81	1.15	690	5.520
**Acetone**	58.08	20.70	2.88	1.820	14.560
**Ethyl butyrate**	116.16	5.10	1.76	80	500
***n*** **-butyraldehyde**	72.11	13.40	2.72	355	2840
**Water**	18.01	80.10	1.85	4.935	20.038
