# Supplementary figures and images for: Advanced tetra amino (ATA-100) cobalt(II) phthalocyanine-based metallo-covalent organic polymer for sensitively detecting volatile organic compounds
Source: Turk J Chem. 2023 Oct 11;47(5):1138–48. doi: 10.55730/1300-0527.3600 (PMC10760821; doi:10.55730/1300-0527.3600)

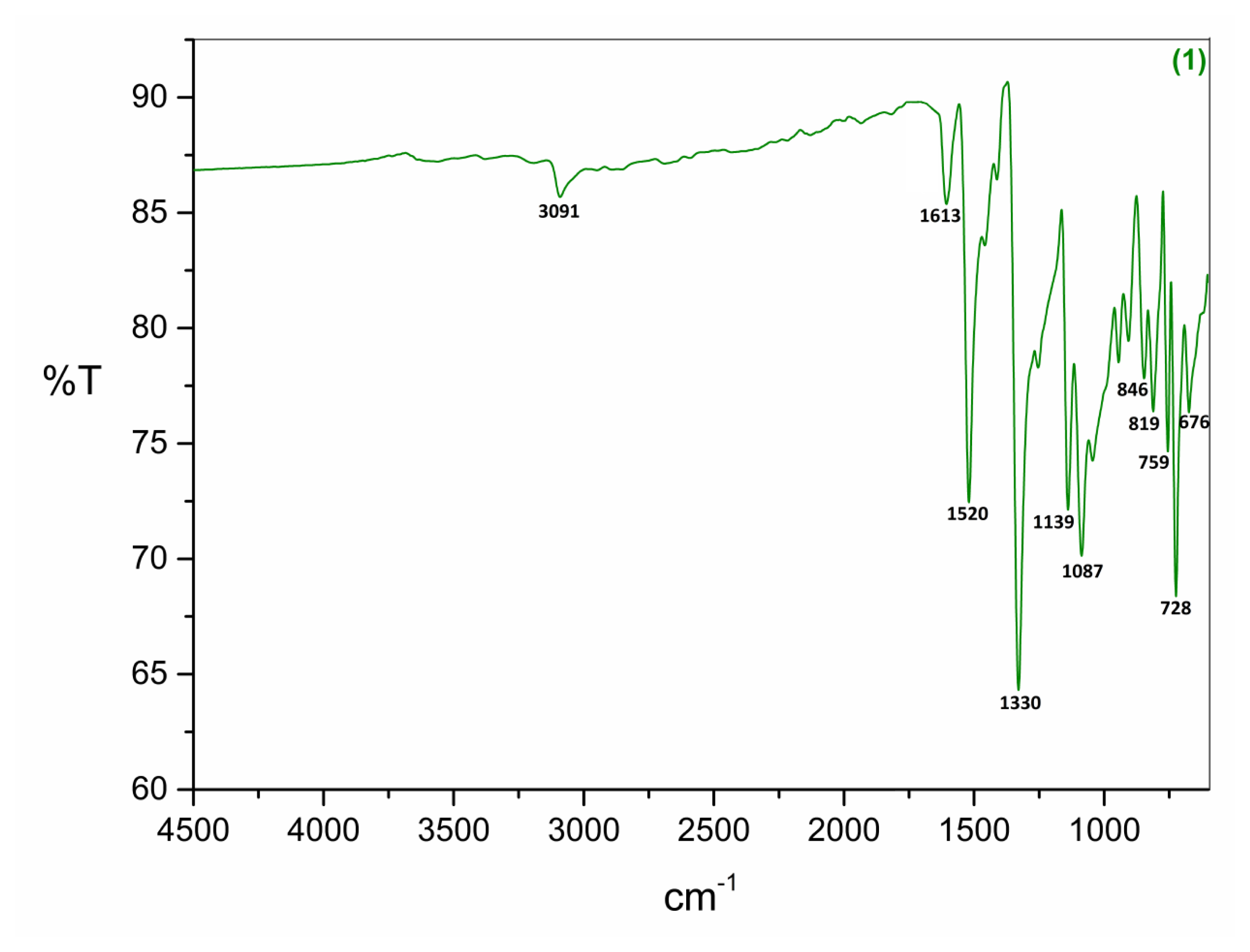

Supplement: Figure S1 — FTIR spectrum of compound 1. [file turkjchem-47-5-1138s1.tif]

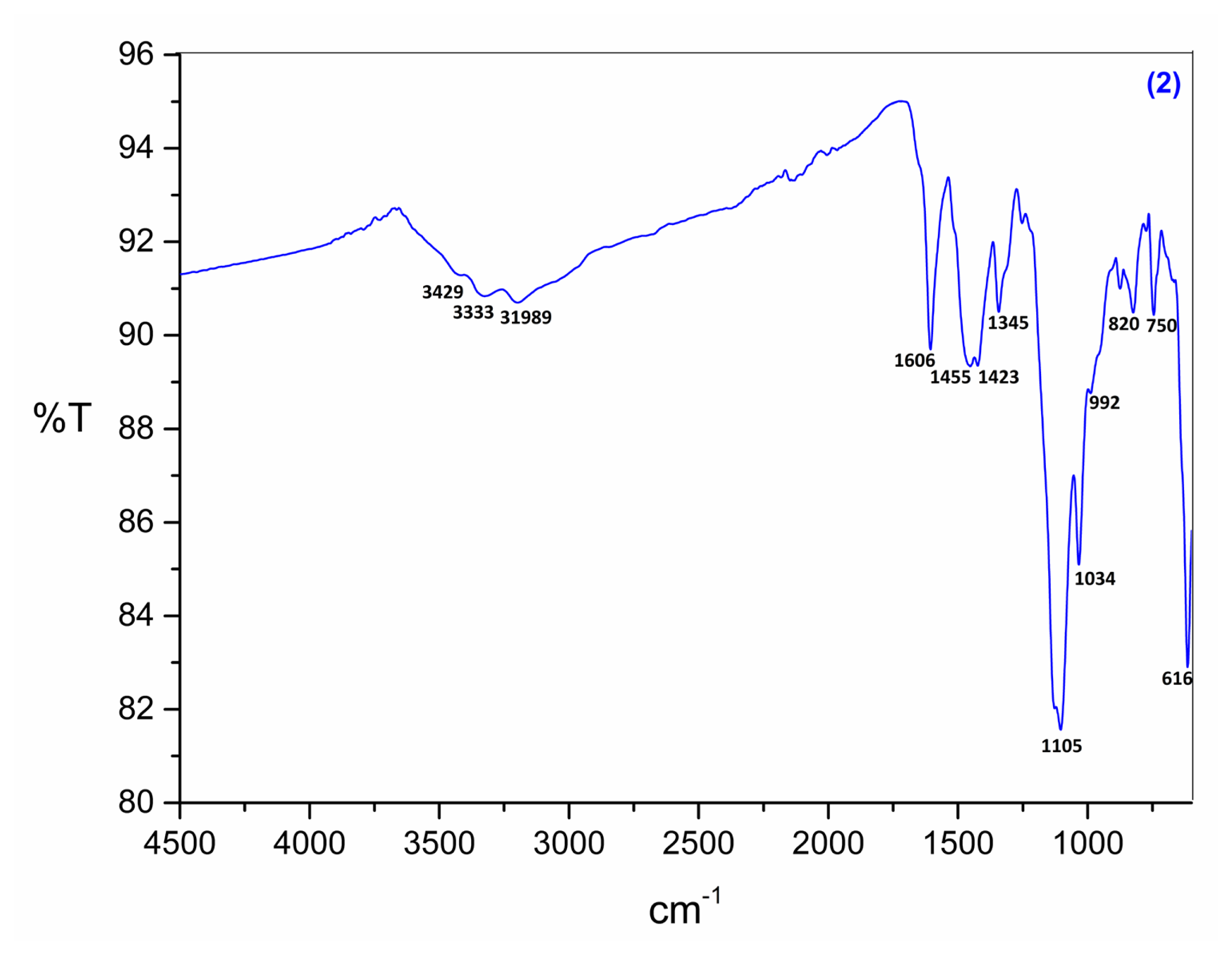

Supplement: Figure S2 — FTIR spectrum of compound 2. [file turkjchem-47-5-1138s2.tif]

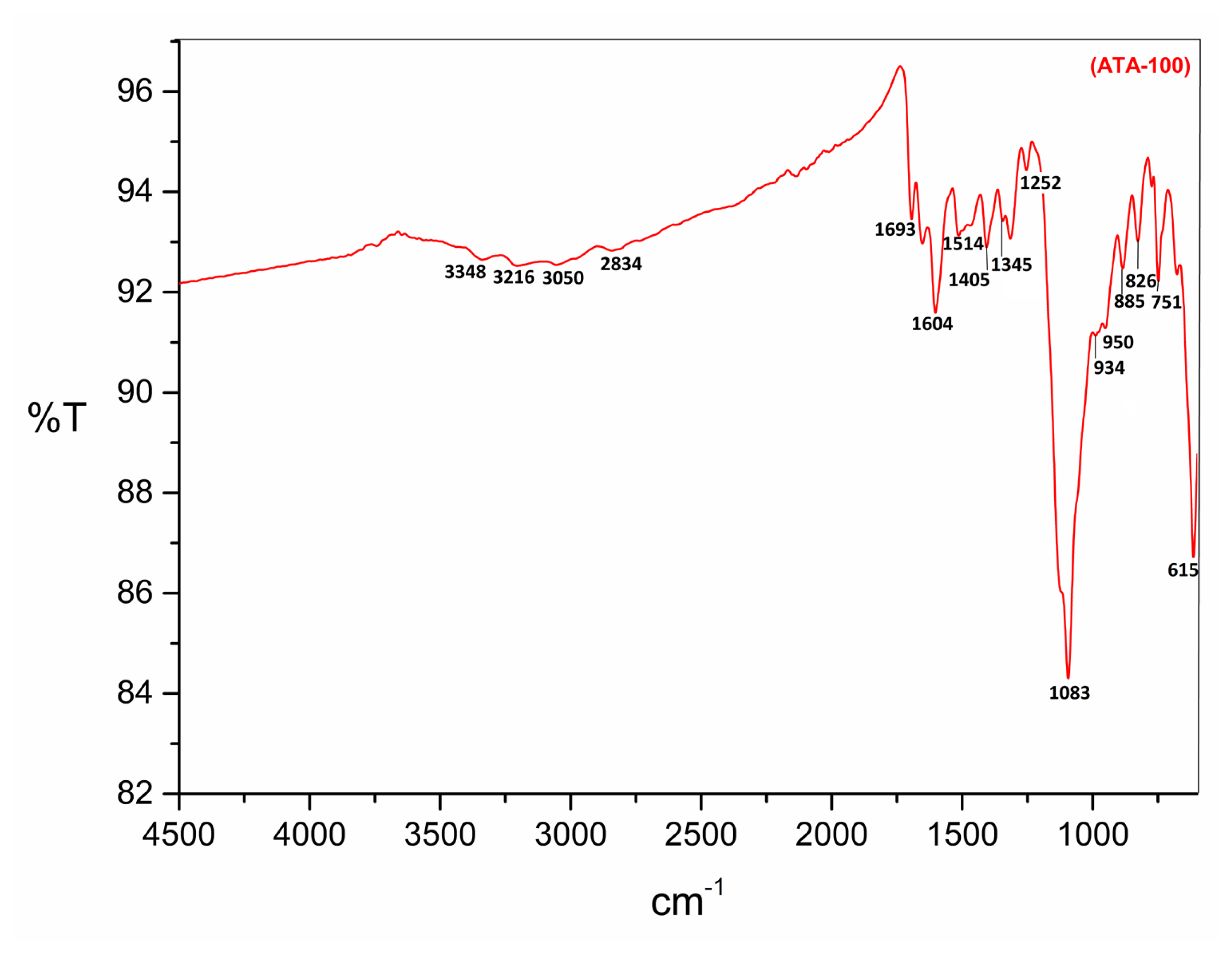

Supplement: Figure S3 — FTIR spectrum of ATA-100. [file turkjchem-47-5-1138s3.tif]

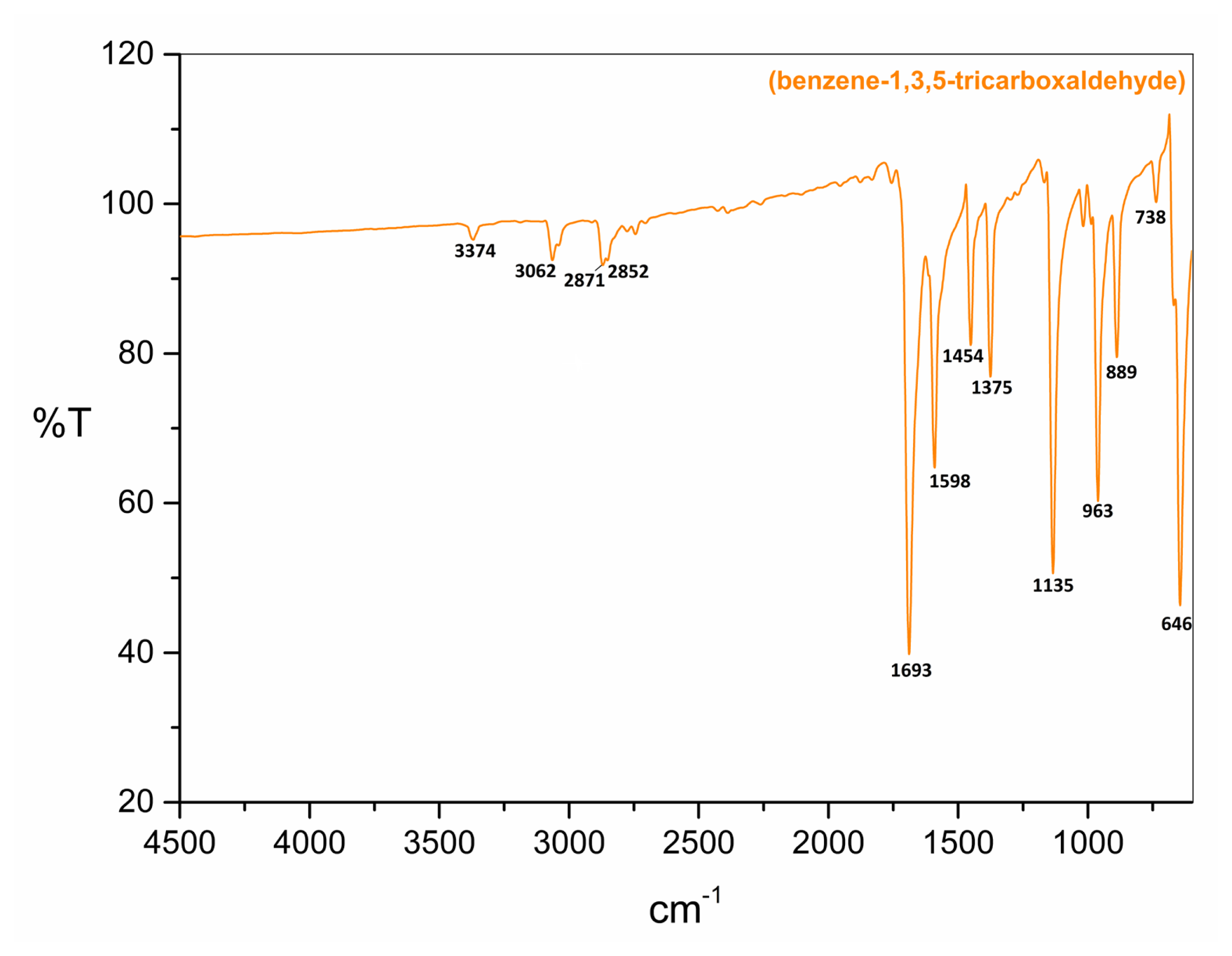

Supplement: Figure S4 — FTIR spectrum of benzene-1,3,5-tricarboxaldehyde. [file turkjchem-47-5-1138s4.tif]

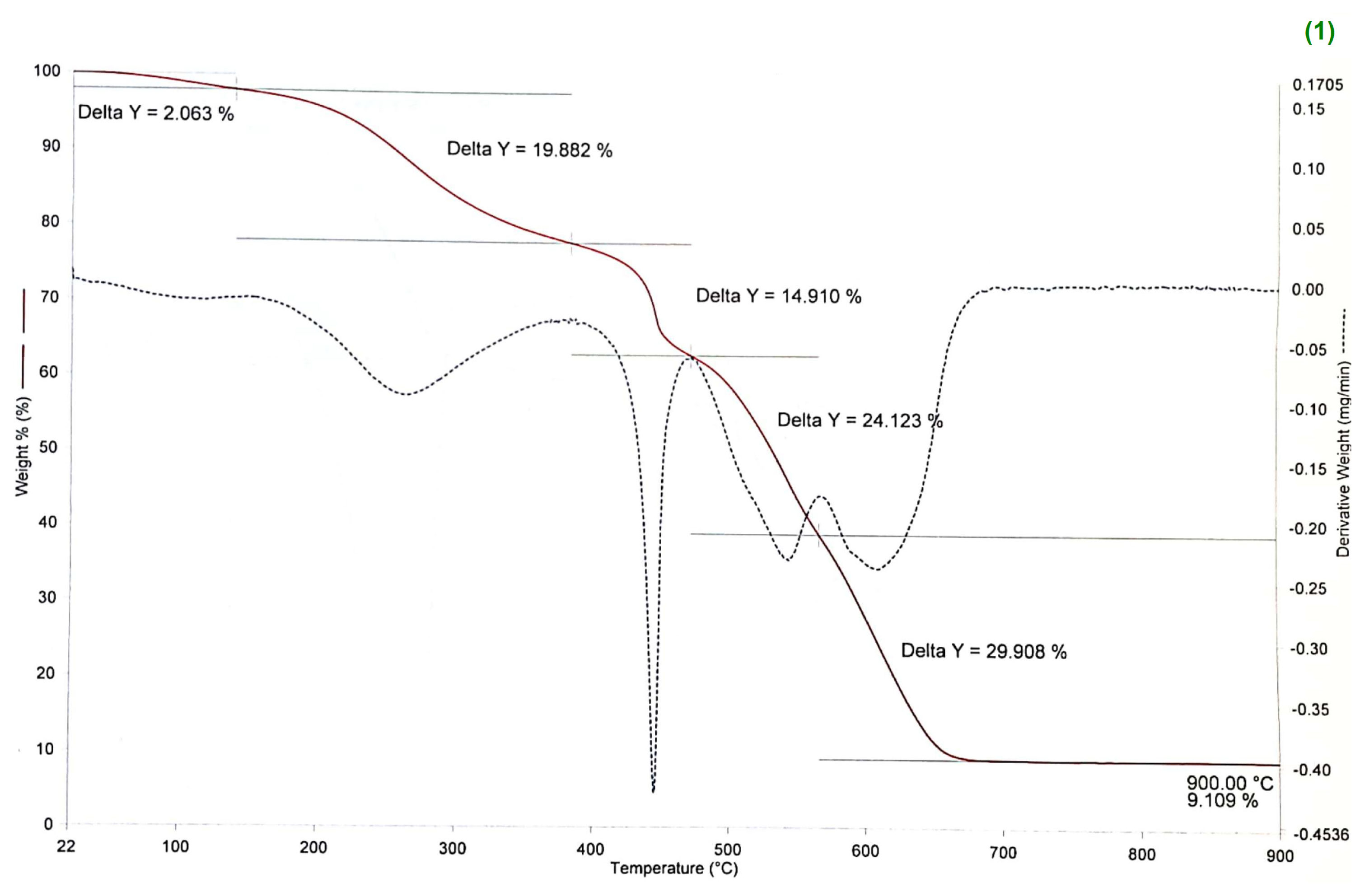

Supplement: Figure S5 — TGA spectrum of compound 1. [file turkjchem-47-5-1138s5.tif]

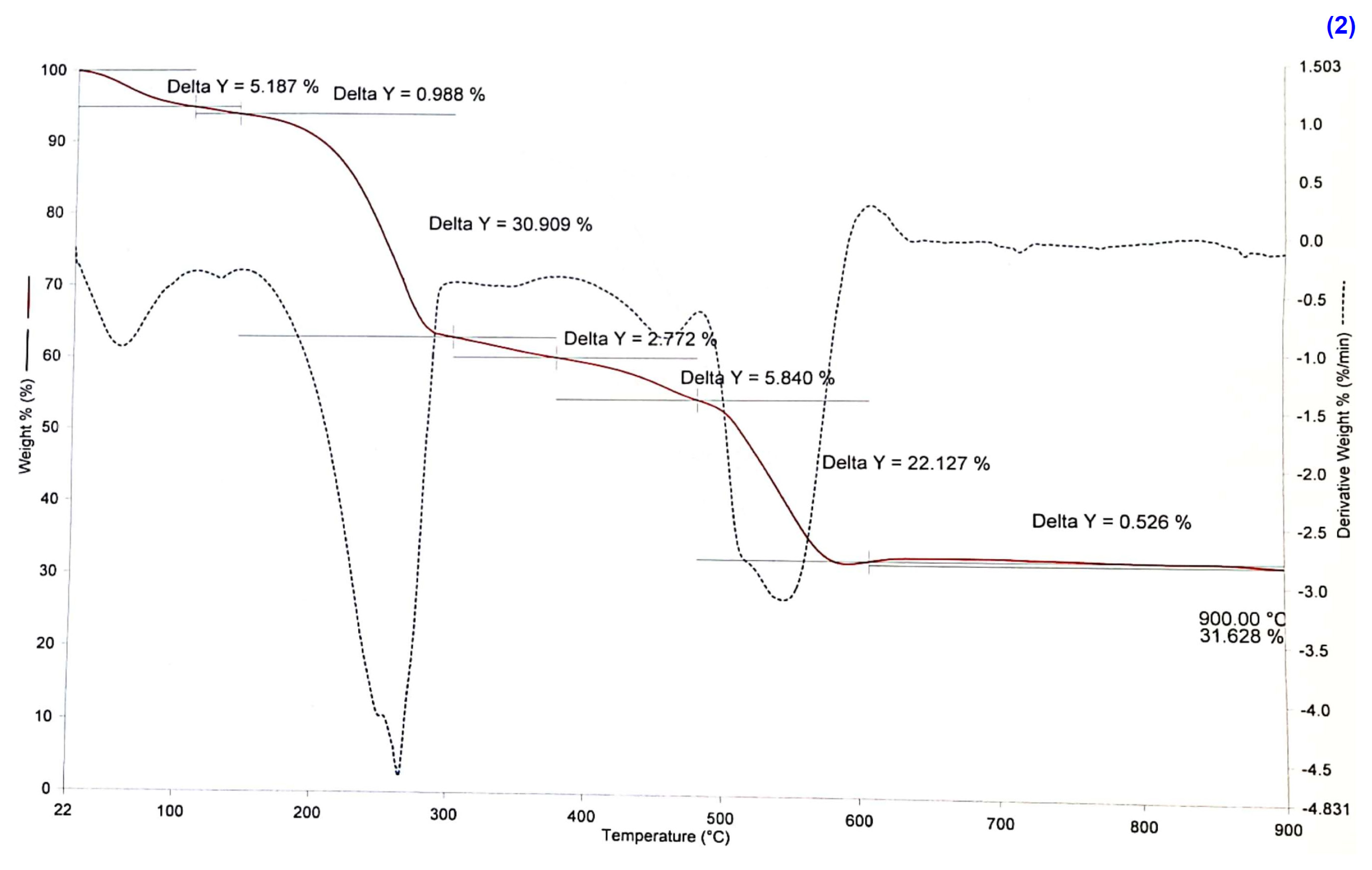

Supplement: Figure S6 — TGA spectrum of compound 2. [file turkjchem-47-5-1138s6.tif]

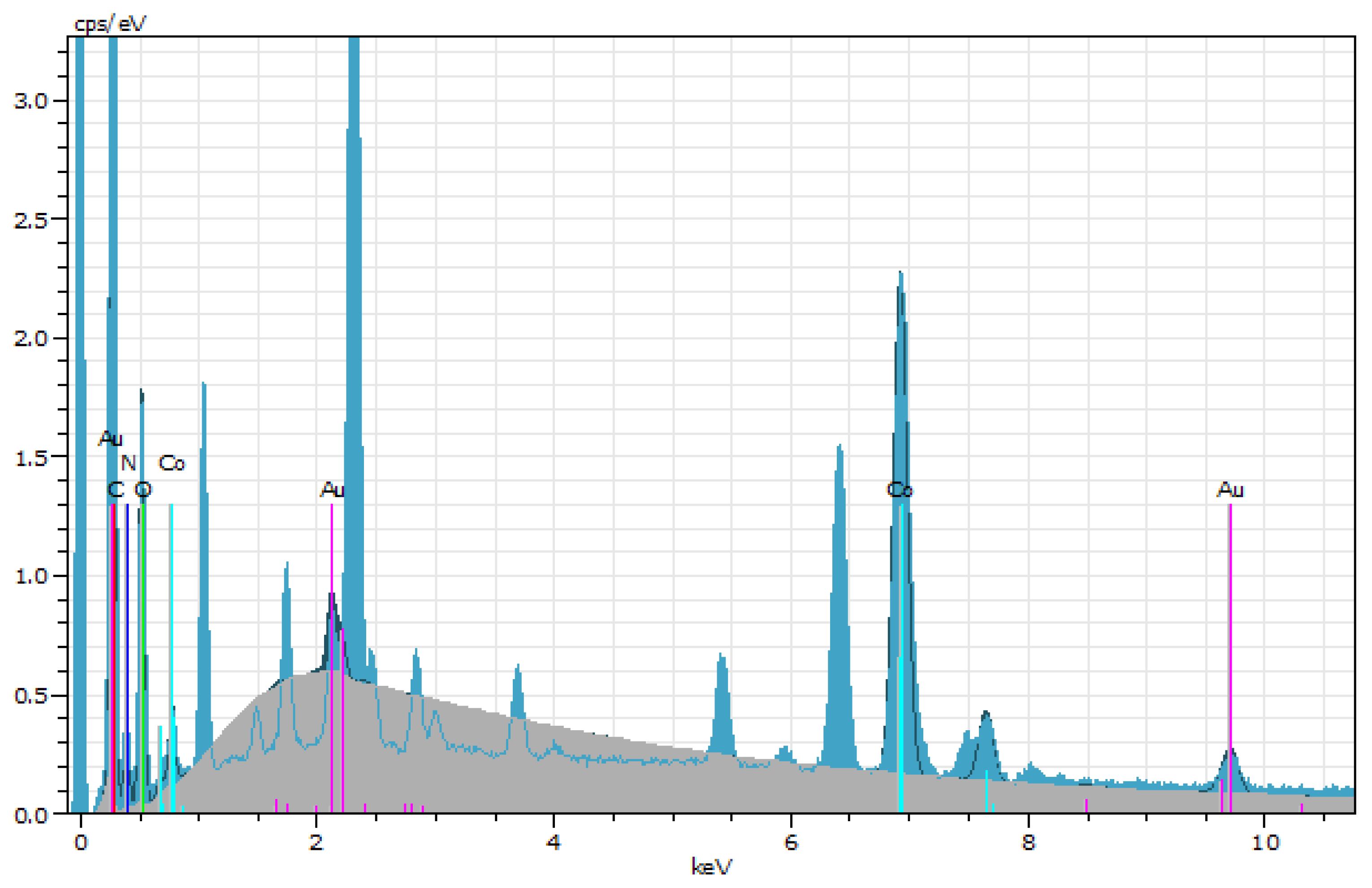

Supplement: Figure S7 — EDS spectrum of ATA-100. [file turkjchem-47-5-1138s7.tif]

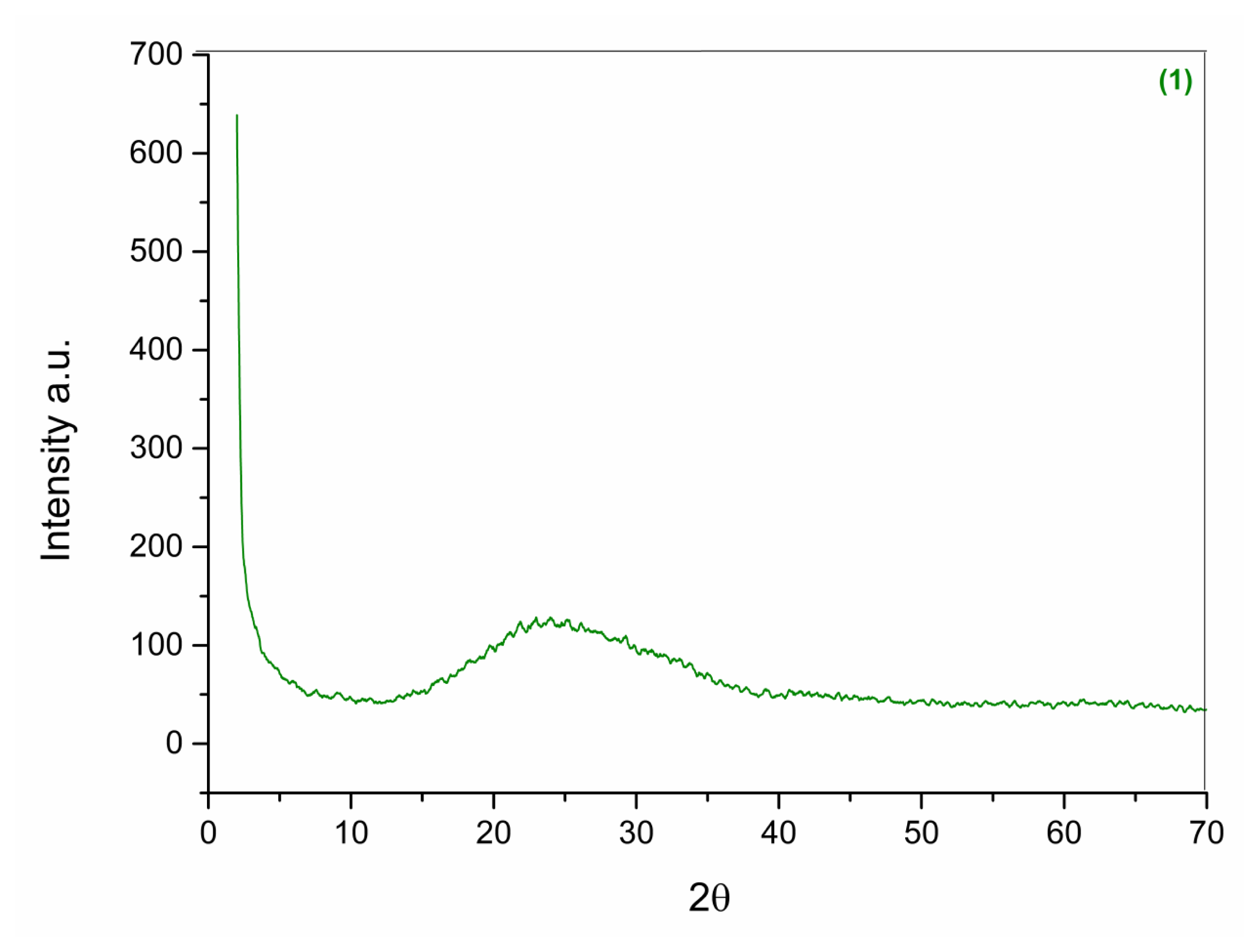

Supplement: Figure S8 — PXRD spectrum of compound 1. [file turkjchem-47-5-1138s8.tif]

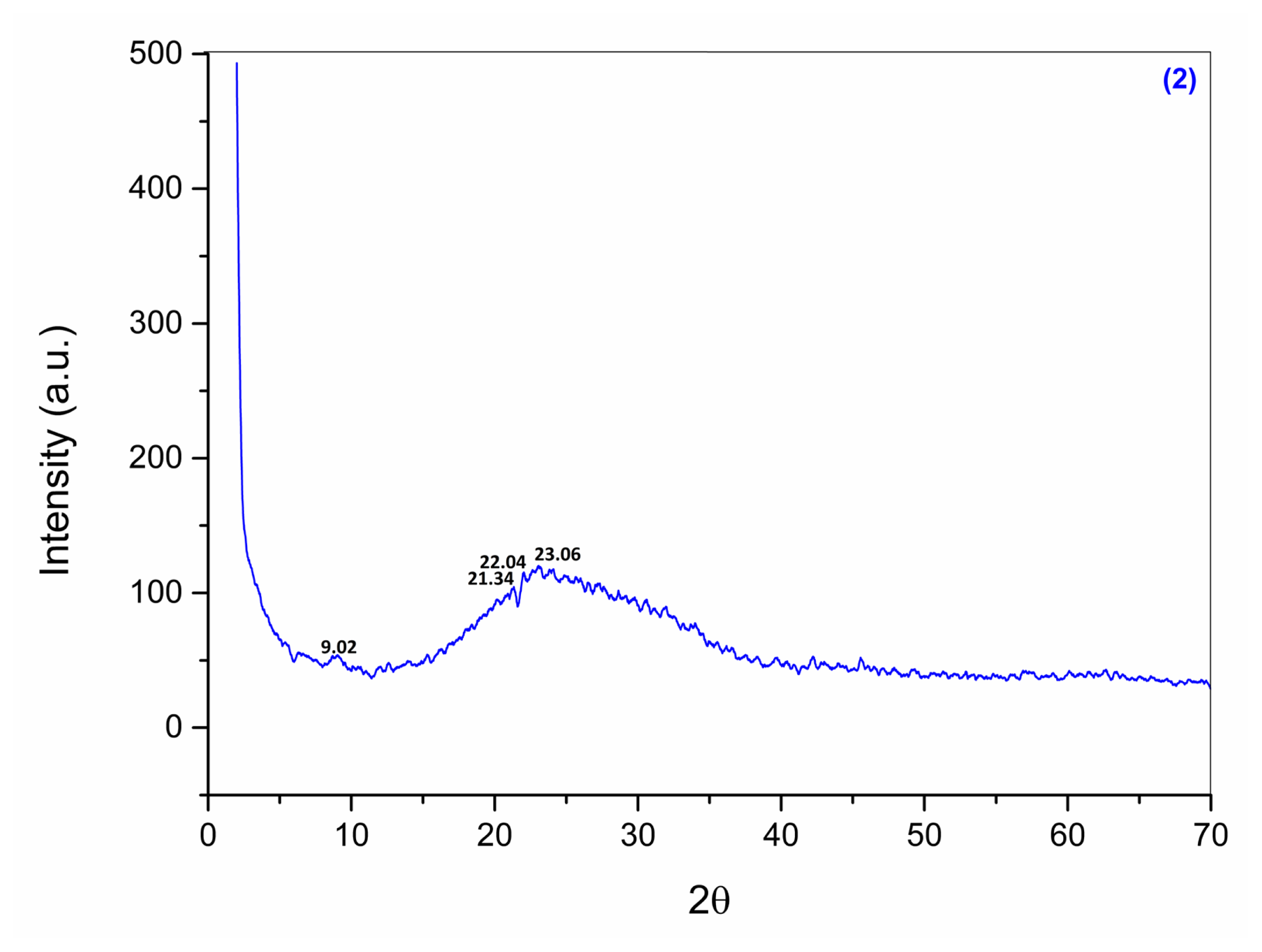

Supplement: Figure S9 — PXRD spectrum of compound 2. [file turkjchem-47-5-1138s9.tif]

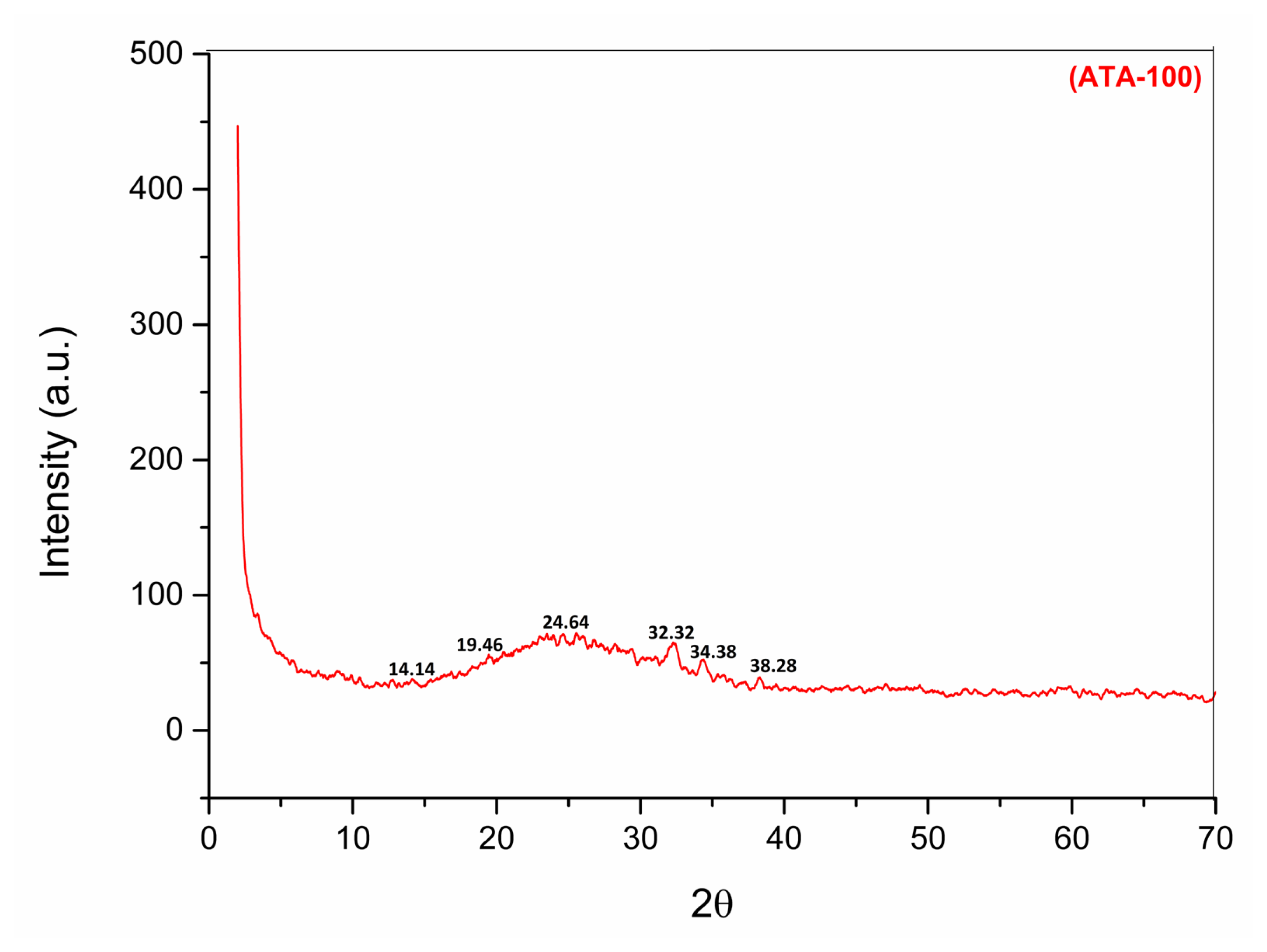

Supplement: Figure S10 — PXRD spectrum of ATA-100. [file turkjchem-47-5-1138s10.tif]

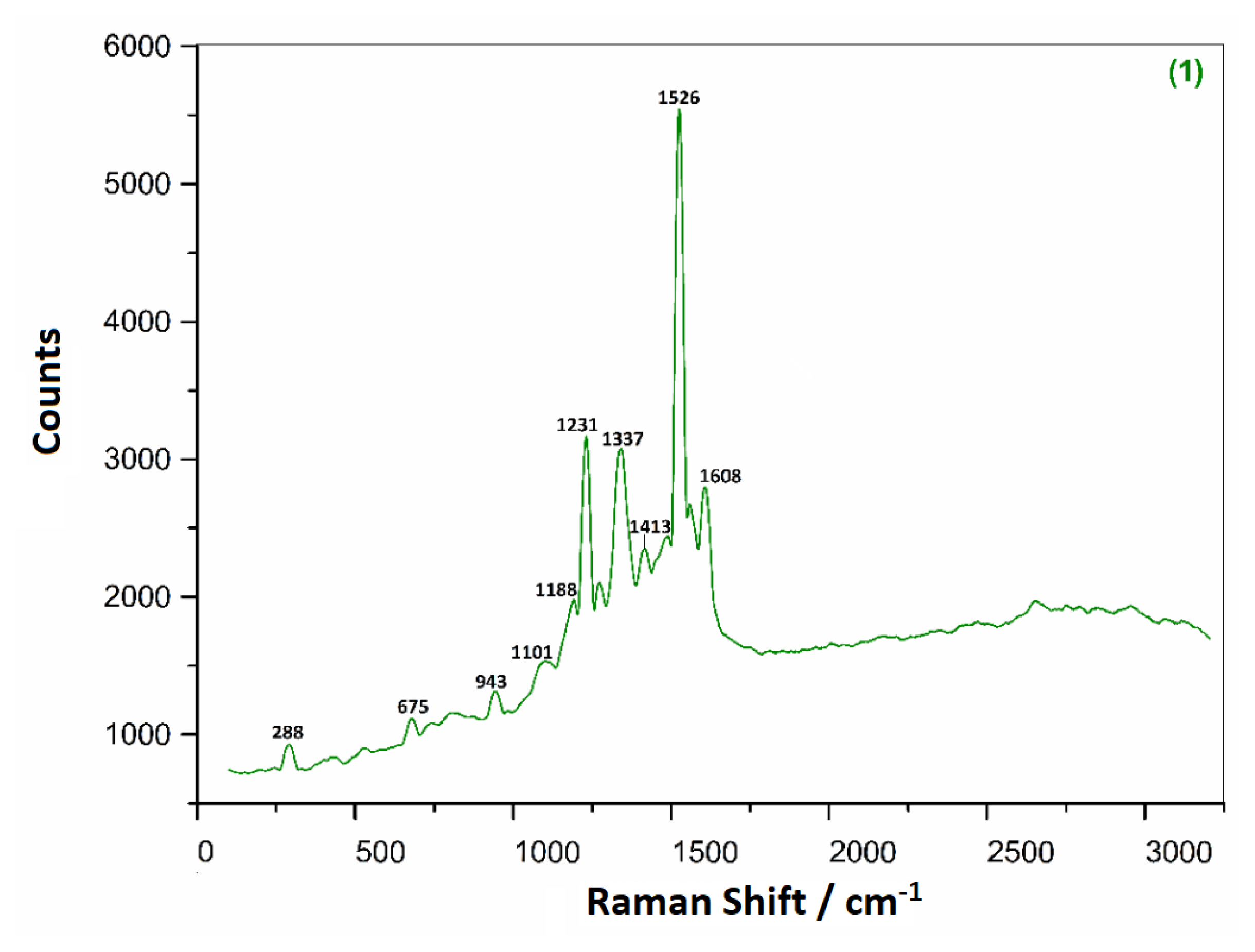

Supplement: Figure S11 — Raman spectrum of compound 1. [file turkjchem-47-5-1138s11.tif]

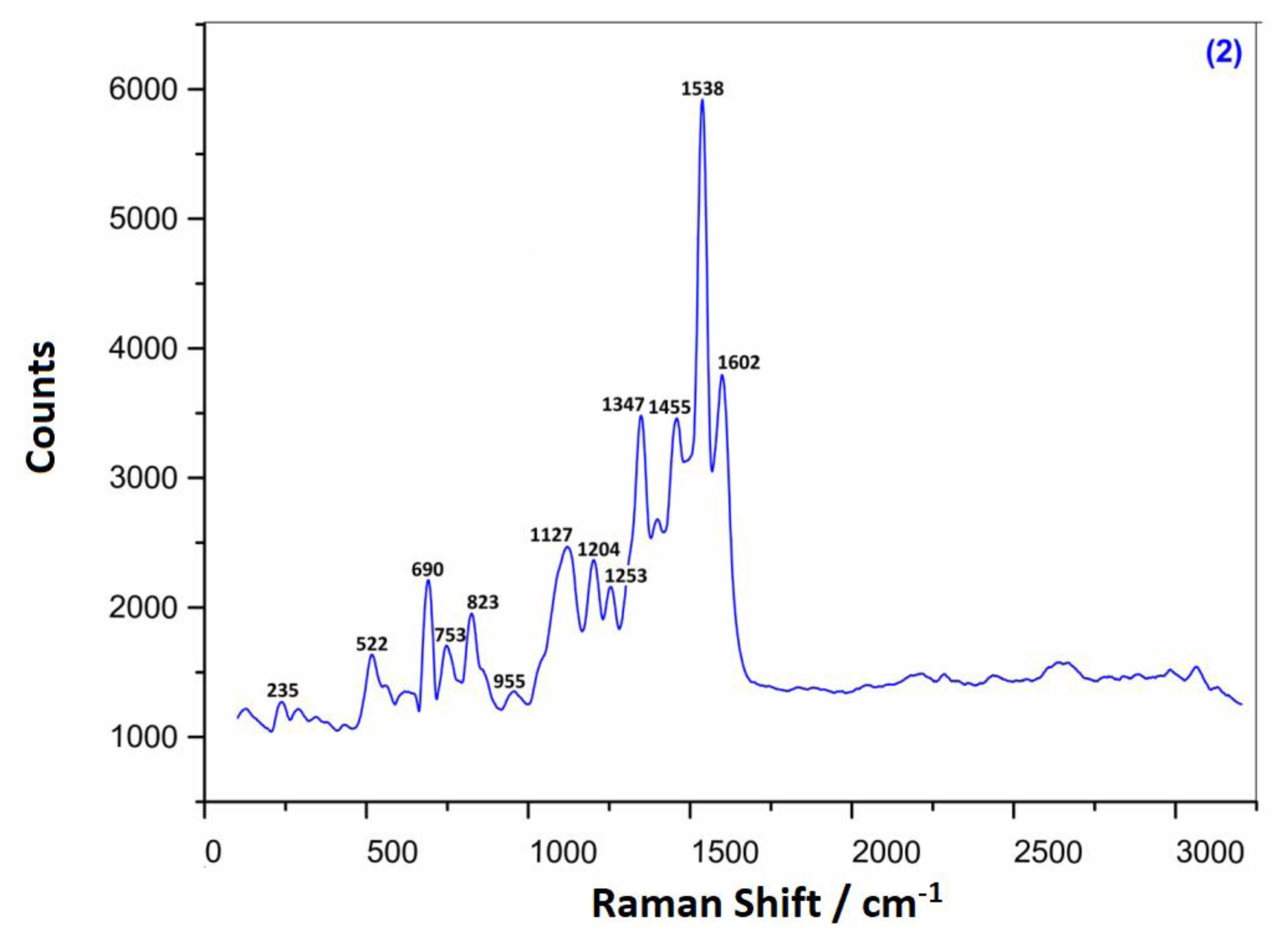

Supplement: Figure S12 — Raman spectrum of compound 2. [file turkjchem-47-5-1138s12.tif]

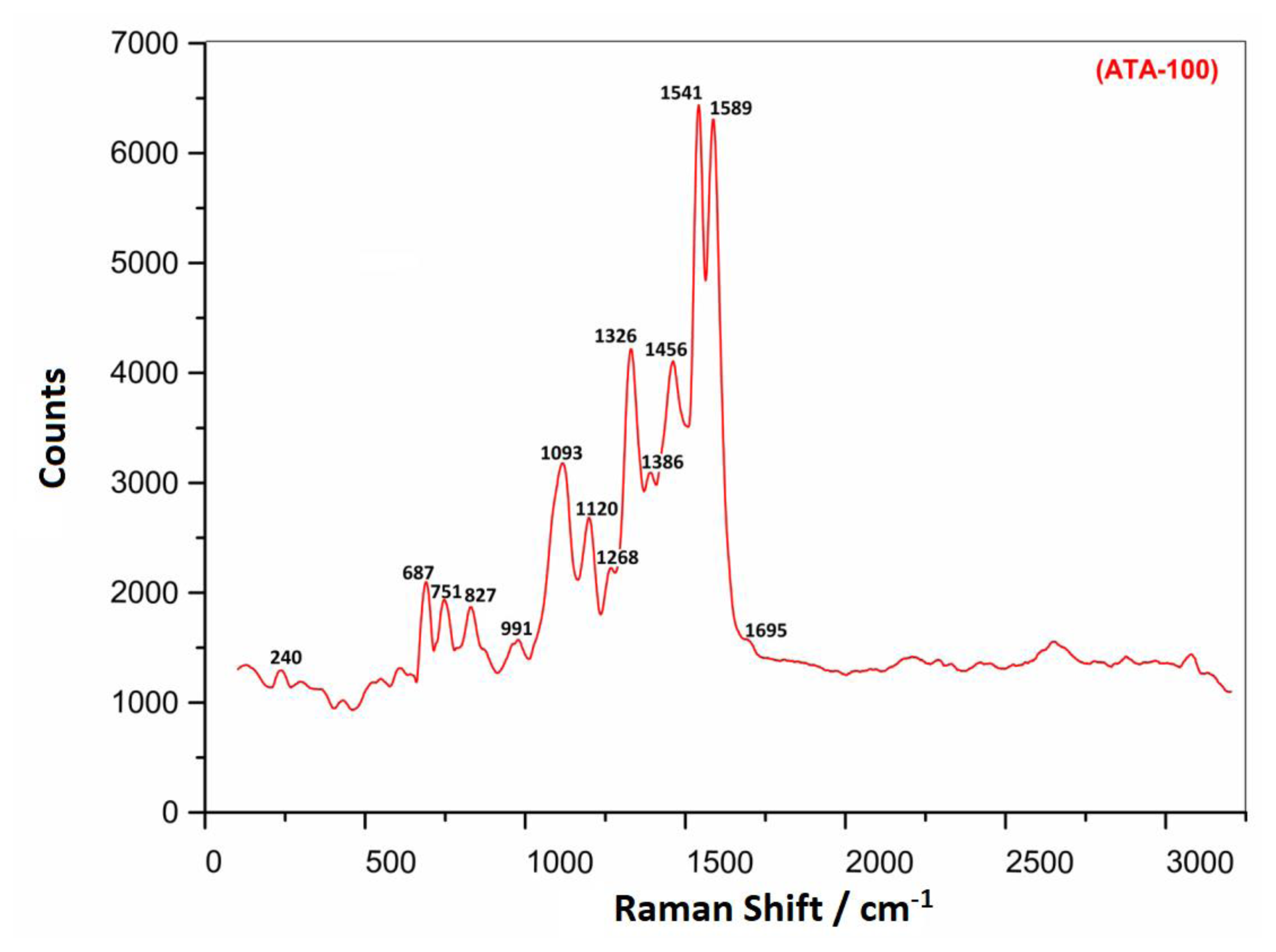

Supplement: Figure S13 — Raman spectrum of ATA-100. [file turkjchem-47-5-1138s13.tif]
